# Association between the *ERCC2* Asp312Asn polymorphism and risk of cancer

**DOI:** 10.18632/oncotarget.17290

**Published:** 2017-04-20

**Authors:** Feifan Xiao, Jian Pu, Qiongxian Wen, Qin Huang, Qinle Zhang, Birong Huang, Shanshan Huang, Aihua Lan, Yuening Zhang, Jiatong Li, Dong Zhao, Jing Shen, Huayu Wu, Yan He, Hongtao Li, Xiaoli Yang

**Affiliations:** ^1^ Medical Scientific Research Center, Guangxi Medical University, Nanning, Guangxi, P.R. China; ^2^ First Clinical Academy, Guangxi Medical University, Nanning, Guangxi, P.R. China; ^3^ Liver and Gall Surgical Department, The Affiliated Hospital of Youjiang Medical College for Nationalities, Baise, Guangxi, P.R. China; ^4^ School of Nursing, The Second Affiliated Hospital of Guilin Medical University, Guilin, Guangxi, P.R. China; ^5^ Guangxi Key Laboratory of Chemistry and Engineering of Forest Products, Guangxi University for Nationalities, Nanning, Guangxi, P.R. China; ^6^ Genetic and Metabolic Central Laboratory, The Maternal and Children Health Hospital of Guangxi, Nanning, Guangxi, P.R. China; ^7^ Department of Cell Biology and Genetics, School of Premedical Sciences, Guangxi Medical University, Nanning, Guangxi, P.R. China; ^8^ Geriatrics Cardiology Division, First Affiliated Hospital of Guangxi Medical University, Nanning, Guangxi, P.R. China

**Keywords:** ERCC2 Asp312Asn, polymorphism, cancer, meta-analysis, trial sequence analysis

## Abstract

Cancer is the leading cause of death in economically developed countries and the second leading cause of death in developing countries. The relationship between genetic polymorphisms and the risk of cancers has been widely researched. Excision repair cross-complementing group 2 (*ERCC2*) gene plays important roles in the nucleotide excision repair pathway. There is contrasting evidence on the association between the *ERCC2* Asp312Asn polymorphism and the risk of cancer. We conducted a comprehensive meta-analysis in order to assess the correlation between these factors. We searched the PubMed, EMBASE, Science Direct, Web of Science, and CNKI databases for studies published from January 1, 2005 to January 1, 2016. Finally, 86 articles with 38,848 cases and 48,928 controls were included in the analysis. The overall analysis suggested a significant association between the *ERCC2* Asp312Asn polymorphism and cancer risk. Furthermore, control source, ethnicity, genotyping method, and cancer type were used for subgroup analysis. The result of a trial sequential analysis indicated that the cumulative evidence is adequate; hence, further trials were unnecessary in the overall analysis for homozygote comparison. In summary, our results suggested that *ERCC2* Asp312Asn polymorphism is associated with increased cancer risk. A significantly increased cancer risk was observed in Asian populations, but not in Caucasian populations. Furthermore, the *ERCC2* Asp312Asn polymorphism is associated with bladder, esophageal, and gastric cancers, but not with breast, head and neck, lung, prostate, and skin cancers, and non-Hodgkin lymphoma. Further multi-center, well-designed studies are required to validate our results.

## INTRODUCTION

Cancer describes a group of diseases characterized by the uncontrolled growth and spread of abnormal cells [1]. It is the leading cause of death in economically developed countries and the second leading cause of death in developing countries [2]. According to statistics, a total of 1,658,370 new cancer cases and 589,430 cancer deaths were projected to occur in the United States in 2015 [3]. In general, cancer is the result of multiple environmental and genetic risk factors, as well as gene-environment interactions [4]. Among genetic factors, genetic and epigenetic mutations, such as aberrant DNA methylation, can lead to carcinogenesis [1].

Recently, the relationship between genetic polymorphisms and the risk of cancer has been widely researched. Among the polymorphic genes, excision repair cross-complementing group 2 (*ERCC2*), also called xeroderma pigmentosum group D (*XPD*), plays important roles in the nucleotide excision repair (NER) pathway [5]. The *ERCC2* gene is located on chromosome 19q13.3, comprises 23 exons, and spans approximately 54,000 base pairs [6]. It encodes an evolutionarily conserved helicase, which has ATP-dependent helicase activity within its multi subunit core transcription factor IIH (TFIIH). The helicase participates in DNA unwinding as part of the NER pathway, and plays an important role in the recognition and repair of structurally unrelated DNA lesions containing bulky adducts and thymidine dimers [7, 8]. Some studies have shown that *ERCC2* polymorphisms may be related to reduced DNA repair due to a possible reduction in its helicase activity [9, 10].

There are two important single nucleotide polymorphisms (SNPs) in the *ERCC2* gene. One is the Lys751Gln polymorphism, which has been shown to be involved in genetic susceptibility to some cancer types. Another common *ERCC2* polymorphism in the coding region is Asp312Asn (rs1799793) [11], which is characterized by a G to A transition at position 312 in exon 10 causing an aspartic acid (Asp) to asparagine amino acid (Asn) exchange [12]. This polymorphism has been widely studied for its association with susceptibility to cancer including brain [13], esophageal [14–16], head and neck [11], bladder [17–19], and breast cancers [20–22]. However, the results reported by these studies were inconsistent.

To provide a comprehensive assessment of and to clarify associations between the *ERCC2* Asp312Asn polymorphisms and the risk of cancer, we performed a meta-analysis of all the eligible case-control studies.

## RESULTS

### Eligible studies

**Figure 1:**
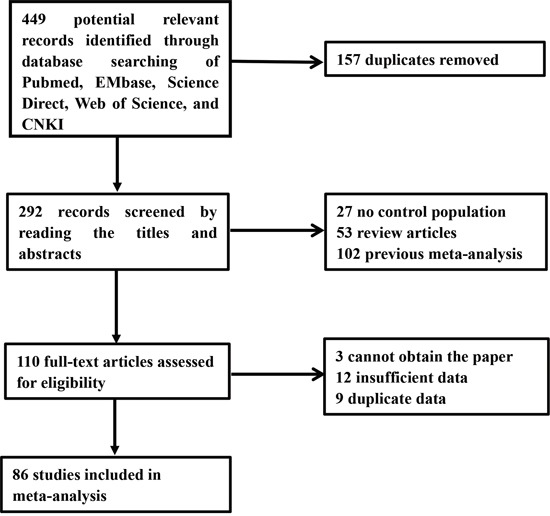
Flow chart showing the selection process for the included studies

A total of 449 articles were reviewed, and eventually 86 articles with 38,848 cases and 48,928 controls met the inclusion criteria. Among these publications, there was 1 osteosarcoma [23], 1 hepatocellular cancer (HCC) [24], 3 oral cancer [25–27], 5 skin cancer [28–32], 5 colorectal cancer [23, 33–36], 6 head and neck cancer [37–42], 6 esophageal cancer [43–48], 6 non-Hodgkin lymphoma [49–54], 6 prostate cancer [55–60], 8 gastric cancer [61–67], 12 bladder cancer [68–79], 14 lung cancer [70, 80–92], and 15 breast cancer [23, 32, 93–105]. The detailed study selection process is shown in Figure 1. Table 1 presents the major characteristics of the 86 articles.

**Table 1:** Characteristics of the case–control studies included in the meta-analyses

First author	Year	Ethnicity	Country^a^	Source of controls	Cancer site	Genotyping method	cases			controls		
							Asp/Asp	Asp/Asn	Asn/Asn	Asp/Asp	Asp/Asn	Asn/Asn
Liu G	2007	Caucasian	USA	HB	esophageal cancer	PCR-RFLP	75	92	16	144	160	32
An	2007	Caucasian	USA	HB	head and neck cancera	PCR-RFLP	330	395	104	370	386	98
Harth	2008	Caucasian	Germany	HB	head and neck cancera	Real-time PCR	113	158	40	101	145	52
Abbasi	2009	Caucasian	Germany	PB	head and neck cancera	Real-time PCR	93	119	34	258	304	82
Ji	2010	Asian	Korea	HB	head and neck cancera	PCR	235	29	0	309	30	3
Gugatschka	2011	Caucasian	Austria	PB	head and neck cancera	TaqMan	116	133	42	171	208	83
Smedby	2006	Caucasian	Sweden	PB	non- Hodgkin lymphoma	PCR	167	211	50	262	255	85
Shen	2006	Caucasian	USA	PB	non- Hodgkin lymphoma	Real-time PCR	199	189	57	226	238	70
Song	2008	Asian	China	HB	non- Hodgkin lymphoma	PCR-RFLP	256	47	4	265	35	3
Baris	2009	Caucasian	Turkey	HB	non- Hodgkin lymphoma	PCR-RFLP	13	16	4	15	27	10
Worrillow	2009	Caucasian	England	PB	non- Hodgkin lymphoma	TaqMan	270	265	79	316	335	79
EI-Din	2013	Caucasian	Egypt	HB	non- Hodgkin lymphoma	PCR-RFLP	30	37	14	38	44	18
Capella G	2008	Mixed	Spain	PB	gastric cancer	PCR-RFLP	110	96	38	444	532	159
Zhou RM	2007	Asians	China	PB	gastric cancer	PCR-RFLP	221	32	0	528	82	2
Lou Y	2006	Asians	China	HB	gastric cancer	PCR-RFLP	189	39	10	176	21	3
Agalliu	2010	Caucasian	USA	PB	prostate cancer	PCR-RFLP	545	575	120	527	528	166
Agalliu	2010	African	USA	PB	prostate cancer	PCR-RFLP	106	31	7	65	15	2
Moreno V	2006	Caucasian	Spain	HB	colorectal cancer	PCR	95	91	100	77	72	63
Hansen RD	2007	Caucasian	Denmark	PB	colorectal cancer	TaqMan	159	191	46	333	354	108
De Ruyck	2007	Caucasian	Belgium	HB	Lung Cancer	PCR-RFLP	44	53	13	49	46	14
Zienolddiny	2006	Caucasian	Norway	PB	Lung Cancer	PCR	119	102	54	120	121	49
Matullo	2006	Caucasian	Europe	PB	Lung Cancer	PCR-RFLP	49	48	19	418	506	170
Hu	2006	Asian	China	HB	Lung Cancer	TaqMan	850	116	4	874	111	1
Shen	2005	Asian	China	PB	Lung Cancer	PCR	109	9	0	99	14	0
Huang	2006	Mixed	USA	NA	Lung Cancer	PCR	301	300	82	301	304	93
Broberg	2005	Caucasian	Sweden	PB	bladder cancer	PCR	16	29	12	61	71	13
Matullo	2005	Caucasian	Italy	HB	bladder cancer	PCR-RFLP and TaqMan	92	153	47	103	155	47
Matullo	2006	Caucasian	European	PB	bladder cancer	TaqMan	48	60	16	418	506	170
Schabath	2005	Mixed	USA	HB	bladder cancer	PCR-RFLP	225	215	57	248	179	50
Andrew	2006	Mixed	USA	PB	bladder cancer	PCR-RFLP	113	145	38	205	251	51
Garcia-Closas	2006	Caucasian	Spain	HB	bladder cancer	PCR	517	474	138	538	467	117
Wu	2006	Caucasian	USA	HB	bladder cancer	PCR-RFLP	264	283	78	283	243	65
Fontana	2008	Caucasian	French	HB	bladder cancer	TaqMan	25	19	7	21	18	6
Chang	2009	Asian	China	HB	bladder cancer	PCR-RFLP	153	98	57	199	67	42
Gangwar	2009	Asian	India	HB	bladder cancer	PCR-RFLP	72	100	34	128	104	18
Mittal	2012	Asian	India	PB	bladder cancer	PCR	78	100	34	128	104	18
Ye	2006	Caucasian	Sweden	PB	esophageal cancer	PCR-RFLP	61	92	24	176	237	57
Tse	2008	Mixed	USA	HB	esophageal cancer	TaqMan	117	150	43	199	206	49
Pan	2009	Caucasian	USA	HB	esophageal cancer	TaqMan	16	20	1	201	185	48
Pan	2009	Caucasian	USA	HB	esophageal cancer	TaqMan	137	163	43	201	185	48
Huang	2012	Asian	China	HB	esophageal cancer	PCR-RFLP	171	42	0	298	60	0
Li	2013	Asian	China	HB	esophageal cancer	PCR-RFLP	342	56	2	351	47	2
Han	2005	Mixed	USA	PB	Skin Cancer	TaqMan	88	99	19	342	373	121
Wang LL	2009	Asian	China	HB	colorectal cancer	PCR-RFLP	132	29	9	176	21	3
Mahimkar MB	2010	Asian	India	NA	oral cancer	PCR-RFLP	23	13	4	23	21	1
Wang Y	2007	Caucasian	USA	HB	oral cancer	PCR and Taqman	50	59	16	140	109	29
Majumder M	2007	Asian	India	HB	oral cancer	PCR	269	208	52	205	146	36
Crew	2007	NA	USA	PB	breast cancer	Taqman	415	478	138	490	454	139
Jorgensen	2007	Caucasian	USA	PB	breast cancer	Taqman	110	128	22	102	142	29
Kuschel	2005	Australian	UK	PB	breast cancer	TaqMan	1529	1530	497	1401	1437	430
Lee	2005	Asian	Korea	HB	breast cancer	PCR	475	50	3	401	41	3
Bernard-Gallon	2008	NA	France	HB	breast cancer	Taqman	403	383	118	458	418	118
Debniak	2006	Polish	Poland	PB	breast cancer	PCR-RFLP	672	785	269	180	252	79
Jakubowska	2010	Polish	Poland	HB	breast cancer	PCR	118	152	44	106	135	49
Mechanic	2006	Caucasian	USA	PB	breast cancer	PCR-RFLP	543	589	130	489	516	128
Mechanic	2006	African-American	USA	PB	breast cancer	PCR-RFLP	564	181	15	517	145	13
Shen	2006	American	USA	PB	breast cancer	Taqman	60	80	16	59	64	30
Smith	2008	Caucasian	USA	HB	breast cancer	PCR	126	137	41	161	188	42
Smith	2008	African-American	USA	HB	breast cancer	PCR	33	14	2	57	16	1
Zhang	2005	Asian	China	PB	breast cancer	PCR-RFLP	89	111	20	119	140	51
Hussien	2012	Caucasian	Egypt	HB	breast cancer	PCR	12	45	43	25	50	25
Jelonek	2010	Mixed	Poland	PB	breast cancer	PCR-RFLP	41	59	21	85	123	23
Wang	2010	Asian	China	PB	breast cancer	PCR-RFLP	624	388	220	925	315	193
Zhou	2012	Asian	Asia	PB	Lung Cancer	PCR-RFLP	85	18	0	85	17	1
Sakoda	2012	Caucasian	USA	PB	Lung Cancer	TaqMan	326	329	89	610	685	182
Qian	2011	Asian	China	PB	Lung Cancer	PCR	464	82	4	497	79	3
Yin	2009	Asian	China	HB	Lung Cancer	PCR-RFLP	246	38	1	255	30	0
Raaschou-Nielsen	2008	Caucasian	Denmark	PB	Lung Cancer	PCR	177	188	59	329	351	107
Chang	2008	Latino-American	USA	PB	Lung Cancer	WGA	60	40	8	192	93	12
Chang	2008	African-American	USA	PB	Lung Cancer	WGA	186	58	3	212	60	5
Yin	2007	Asian	China	HB	Lung Cancer	PCR-RFLP	200	1	0	170	0	1
Lopez-Cima	2007	Caucasian	Spain	HB	Lung Cancer	PCR-RFLP	240	221	55	260	230	43
Han	2005	Mixed	USA	PB	Skin Cancer	TaqMan	104	149	32	342	373	121
Han	2005	Mixed	USA	PB	Skin Cancer	TaqMan	128	115	37	342	373	121
Lovatt	2005	Caucasian	UK	PB	Skin Cancer	PCR-RFLP	224	219	66	151	163	65
Li	2006	Mixed	USA	HB	Skin Cancer	PCR	242	290	70	273	259	71
Millikan	2006	Caucasian	USA	PB	Skin Cancer	PCR	1039	1098	162	1039	1098	260
Debniak	2006	Polish	Poland	mixed	Skin Cancer	PCR	168	188	69	492	597	173
Bau	2007	Asian	Taiwan	HB	prostate cancer	PCR	62	39	22	310	106	63
Mandal	2010	Asian	India	PB	prostate cancer	PCR	76	56	39	99	81	20
Lavende	2010	African	America	HB	prostate cancer	PCR and Taqman	146	39	5	510	116	5
Dhillon	2011	Caucasian	Australia	NA	prostate cancer	PCR-RFLP	71	37	8	80	42	10
Yuan T	2011	Asian	China	HB	gastric Cancer	PCR	156	18	16	133	35	12
Chen Z	2011	Asian	China	HB	gastric Cancer	PCR-RFLP	75	118	15	220	111	8
Zhang CZ	2009	Asian	China	HB	gastric Cancer	PCR-RFLP	75	117	15	132	72	8
Ruzzo A	2007	Caucasian	Italy	HB	gastric Cancer	PCR-RFLP	23	26	20	41	67	13
Deng Sl	2010	Asian	China	HB	gastric Cancer	PCR	132	15	13	118	31	11
Wu JS	2014	Asian	China	HB	HCC	PCR	138	58	22	181	70	26
Sambuddha	2015	Asian	Northeast India	NA	head and neck cancer	PCR	32	40	8	57	31	4
Benjamin	2015	Mexican	Mexica	HB	osteosarcoma	PCR	21	3	4	68	8	21
Benjamin	2015	Mexican	Mexica	HB	colorectal cancer	PCR	74	26	8	81	23	15
Benjamin	2015	Mexican	Mexica	HB	breast cancer	PCR	54	9	8	54	1	19
Min Ni	2014	Asian	China	HB	colorectal cancer	Real-time PCR	182	26	5	210	27	3
Volha P. Ramaniuk	2014	Belarusians	Belarus	HB	bladder cancer	PCR-RFLP	99	178	56	128	169	71
Aneta Mirecka	2014	Polish	Poland	PB	prostate cancer	real-time PCR	199	249	124	377	218	32

^a^ Country of first author.

### Meta-analysis

#### Overall analysis

In the dominant model, increased cancer risk was found with an odds ratio (OR) of 1.110 (95% confidence interval [CI] 1.078-1.143, P<0.01). In the recessive model, significantly increased risk was determined with an OR of 1.059 (95% CI 1.013-1.108, P<0.01). Furthermore, when the homozygote and heterozygote comparisons were performed, increased risk was identified, with an OR of 1.103 (95% CI 1.052-1.157, P<0.01), and an OR of 1.106 (95% CI 1.072-1.141, P<0.01), respectively. Overall, the results of our meta-analysis showed a significant association between the *ERCC2* polymorphism and cancer risk (Table 2).

**Table 2:** Results of overall and stratified meta-analyses

Model (Comparison)	Subgroup	No. of trials	I^2^(%)	*P*^a^	Fixed	Random	*P* for bias
homozygote comparison (Asn/Asn vs. Asp/Asp)	Total	95	68.3	0	1.103(1.052,1.157)	1.170(1.060,1.293)	0.079
	PB	41	79.8	0	1.037(0.977,1.101)	1.074(0.922,1.250)	0.53
	HB	49	39	0.004	1.249(1.149,1.358)	1.283(1.135,1.450)	0.462
	Asia	30	48.3	0.003	1.664(1.461,1.894)	1.734(1.371,2.192)	0.961
	Caucasian	37	50.8	0	0.964(0.899,1.034)	1.019(0.913,1.137)	0.041
	PCR	29	65	0	1.041(0.951,1.140)	1.175(0.983,1.404)	0.054
	PCR-RFLP	38	62.5	0	1.160(1.068,1.260)	1.238(1.053,1.455)	0.054
	Taqman	18	24.8	0.163	1.003(0.921,1.093)	0.983(0.878,1.100)	0.16
	Bladder cancer	12	56.4	0.008	1.370(1.198,1.566)	1.446(1.160,1.803)	0.191
	Breast cancer	18	66.6	0	1.098(1.009,1.194)	1.042(0.871,1.246)	0.543
	Esophageal cancer	7	0	0.62	1.219(0.945,1.571)	1.243(0.962,1.608)	0.074
	Gastric cancer	8	65.3	0.005	1.517(1.167,1.972)	1.876(1.105,3.186)	0.258
	Head and neck cancer	6	52.4	0.062	0.993(0.814,1.212)	0.989(0.707,1.384)	0.909
	Lung Cancer	16	0	0.533	1.043(0.901,1.207)	1.042(0.899,1.207)	0.386
	Prostate cancer	7	93.5	0	1.570(1.314,1.874)	2.038(0.848,4.894)	0.419
	Skin Cancer	7	59.9	0.021	0.784(0.689,0.893)	0.818(0.657,1.020)	0.448
	Non- Hodgkin lymphoma	6	0	0.782	0.998(0.811,1.229)	1.000(0.812,1.231)	0.505
heterozygote comparison (Asp/Asn vs. Asp/Asp)	Total	95	61.1	0	1.106(1.072,1.141)	1.133(1.072,1.198)	0.111
	PB	41	64.7	0	1.061(1.020,1.104)	1.064(0.988,1.146)	0.889
	HB	49	53.9	0	1.205(1.143,1.270)	1.229(1.128,1.339)	0.329
	Asia	30	71.8	0	1.373(1.275,1.480)	1.287(1.105,1.499)	0.096
	Caucasian	37	0	0.801	1.034(0.988,1.083)	1.034(0.987,1.082)	0.526
	PCR	29	44.2	0.006	1.057(0.996,1.121)	1.076(0.982,1.180)	0.281
	PCR-RFLP	38	70	0	1.187(1.126,1.251)	1.203(1.081,1.338)	0.745
	Taqman	18	14.5	0.28	1.030(0.974,1.090)	1.039(0.973,1.109)	0.348
	Bladder cancer	12	31.2	0.142	1.235(1.128,1.353)	1.265(1.125,1.423)	0.231
	Breast cancer	18	70.7	0	1.086(1.025,1.149)	1.101(0.972,1.248)	0.42
	Esophageal cancer	7	0	0.994	1.213(1.051,1.401)	1.213(1.051,1.401)	0.932
	Gastric cancer	8	91.1	0	1.209(1.038,1.409)	1.066(0.614,1.848)	0.491
	Head and neck cancer	6	27.4	0.229	1.114(0.977,1.271)	1.121(0.950,1.323)	0.334
	Lung Cancer	16	0	0.808	1.000(0.918,1.090)	1.001(0.918,1.091)	0.294
	Prostate cancer	7	78.4	0	1.281(1.140,1.440)	1.297(0.965,1.743)	0.879
	Skin Cancer	7	36.5	0.15	1.018(0.938,1.105)	1.023(0.913,1.146)	0.868
	Non- Hodgkin lymphoma	6	27.7	0.227	1.038(0.907,1.187)	1.047(0.881,1.244)	0.938
dominant model((Asn/Asn+Asp/Asn) vs. Asp/Asp)	Total	95	69.3	0	1.110(1.078,1.143)	1.143(1.078,1.212)	0.126
	PB	41	75.9	0	1.060(1.021,1.101)	1.067(0.981,1.160)	0.754
	HB	49	56.6	0	1.217(1.158,1.278)	1.237(1.139,1.343)	0.587
	Asia	30	73.4	0	1.416(1.321,1.518)	1.336(1.153,1.547)	0.13
	Caucasian	37	3.2	0.414	1.020(0.976,1.065)	1.021(0.976,1.068)	0.102
	PCR	29	47.4	0.003	1.053(0.996,1.113)	1.091(0.999,1.191)	0.137
	PCR-RFLP	38	74.5	0	1.191(1.133,1.251)	1.216(1.091,1.356)	0.647
	Taqman	18	11.5	0.317	1.026(0.972,1.082)	1.028(0.968,1.093)	0.908
	Bladder cancer	12	50.2	0.024	1.266(1.162,1.379)	1.309(1.148,1.494)	0.242
	Breast cancer	17	73.4	0	1.091(1.034,1.151)	1.083(0.958,1.223)	0.962
	Esophageal cancer	7	0	0.989	1.214(1.057,1.394)	1.214(1.057,1.394)	0.236
	Gastric cancer	8	90.7	0	1.277(1.106,1.474)	1.229(0.745,2.027)	0.88
	Head and neck cancer	6	50.7	0.071	1.091(0.963,1.236)	1.104(0.908,1.343)	0.493
	Lung Cancer	15	0	0.763	1.010(0.931,1.097)	1.010(0.931,1.097)	0.474
	Prostate cancer	7	89.8	0	1.353(1.213,1.509)	1.407(0.951,2.081)	0.71
	Skin Cancer	7	37.6	0.142	0.968(0.895,1.046)	0.978(0.877,1.090)	0.682
	Non- Hodgkin lymphoma	6	9.4	0.356	1.033(0.909,1.173)	1.035(0.901,1.189)	0.932
recessive model (Asn/Asn vs. (Asp/Asp+Asp/Asn))	Total	95	62.7	0	1.059(1.013,1.108)	1.108(1.016,1.208)	0.098
	PB	41	76.4	0	1.010(0.954,1.069)	1.044(0.914,1.192)	0.501
	HB	49	30.6	0.025	1.157(1.070,1.252)	1.178(1.059,1.310)	0.481
	Asia	30	35.8	0.032	1.445(1.275,1.637)	1.515(1.240,1.852)	0.668
	Caucasian	37	52.2	0	0.954(0.894,1.019)	1.006(0.906,1.115)	0.055
	PCR	29	64.2	0	1.022(0.939,1.113)	1.131(0.959,1.335)	0.107
	PCR-RFLP	38	53	0	1.087(1.006,1.175)	1.147(1.002,1.314)	0.152
	Taqman	18	28.8	0.123	0.987(0.911,1.609)	0.958(0.859,1.069)	0.082
	Bladder cancer	12	48.6	0.029	1.225(1.080,1.389)	1.271(1.052,1.536)	0.189
	Breast cancer	17	60.1	0.001	1.062(0.981,1.149)	1.018(0.874,1.186)	0.421
	Esophageal cancer	7	0	0.615	1.102(0.869,1.398)	1.130(0.888,1.437)	0.086
	Gastric cancer	8	39	0.119	1.563(1.215,2.011)	1.739(1.190,2.541)	0.341
	Head and neck cancer	6	35.4	0.171	0.951(0.790,1.144)	0.944(0.729,1.223)	0.815
	Lung Cancer	15	0	0.806	1.046(0.910,1.203)	1.046(0.910,1.203)	0.495
	Prostate cancer	7	92.4	0	1.406(1.186,1.667)	1.851(0.846,4.050)	0.357
	Skin Cancer	7	63.4	0.012	0.781(0.691,0.883)	0.810(0.653,1.006)	0.557
	Non- Hodgkin lymphoma	6	0	0.619	0.987(0.813,1.200)	0.989(0.814,1.203)	0.646

^a^ *P* for heterogeneity.

#### Subgroup analysis

In order to evaluate the effects of specific study characteristics on the association between the *ERCC2* polymorphism and cancer risk, we performed subgroup analysis if there were 6 or more studies. The ORs and 95% CIs were obtained from the subgroups of control source, ethnicity, genotyping method, and type of cancer. For control source subgroup, we found a significant association between the *ERCC2* polymorphism and cancer risk when the source of the controls was hospital-based (HB). Meanwhile, when the studies recruited population-based (PB) control, no association was found. For ethnicity, no significant association was detected in Caucasians, but significant associations were observed in Asians. When stratified according to the genotyping method, significant associations were observed when the method was polymerase chain reaction restriction fragment length polymorphism (PCR-RFLP). By comparison, no relationship was found when the methods used were PCR and TaqMan assay. According to the type of cancer, the *ERCC2* polymorphism was associated with a significantly higher risk of bladder cancer. In contrast, we observed no association between this polymorphism and breast cancer. Similarly, the results of subgroups of other cancers indicated no association with the *ERCC2* polymorphism, including head and neck, lung, prostate, and skin cancers and non-Hodgkin lymphoma. For the esophageal cancer group, a significant association was obtained in the heterozygote comparison, but not in the homozygote comparison and the recessive model. In the group with gastric cancer, the *ERCC2* polymorphism was confirmed to increase the risk of cancer in the homozygote comparison and the recessive model, but not in the heterozygote comparison and the dominant model. The detailed results are shown in Table 2.

#### Test of heterogeneity

High heterogeneity was observed after the data were pooled (homozygote comparison: *P* for heterogeneity = 0, I^2^ = 68.3%). As shown in Table 2, when the subjects were stratified on the basis of the control source, high heterogeneity remained with PB controls (homozygote comparison: *P* for heterogeneity = 0, I^2^ = 79.8%). Additionally, in analyses of ethnicity, moderate heterogeneity was found in Asian studies (homozygote comparison: *P* for heterogeneity = 0.003, I^2^ = 48.3%), and high heterogeneity was found in Caucasian studies (homozygote comparison: *P* for heterogeneity = 0, I^2^ = 50.8%). Moreover, in analyses of genotyping methods, low heterogeneity was detected in the TaqMan group (homozygote comparison: *P* for heterogeneity = 0.163, I^2^ = 24.8%), but high heterogeneity was found in the PCR (homozygote comparison: *P* for heterogeneity = 0, I^2^ = 65%) and PCR-RFLP groups (homozygote comparison: *P* for heterogeneity = 0, I^2^ = 62.5%). Furthermore, heterogeneity was not detected in esophageal cancer studies (homozygote comparison: *P* for heterogeneity = 0.62, I^2^ = 0.0%), lung cancer studies (homozygote comparison: *P* for heterogeneity = 0.533, I^2^ = 0.0%), and non-Hodgkin lymphoma studies (homozygote comparison: *P* for heterogeneity = 0.782, I^2^ = 0.0%). Nonetheless, high heterogeneity was still present in studies of prostate cancer (homozygote comparison: *P* for heterogeneity = 0, I^2^ = 93.5%), bladder cancer (homozygote comparison: *P* for heterogeneity = 0.008, I^2^ = 56.4%), breast cancer (homozygote comparison: *P* for heterogeneity = 0, I^2^ = 66.6%), gastric cancer (homozygote comparison: *P* for heterogeneity = 0.005, I^2^ = 65.3%), head and neck cancer (homozygote comparison: *P* for heterogeneity = 0.062, I^2^ = 52.4%), and skin cancer (homozygote comparison: *P* for heterogeneity = 0.021, I^2^ = 59.9%).

#### Publication bias and sensitivity analysis

**Figure 2:**
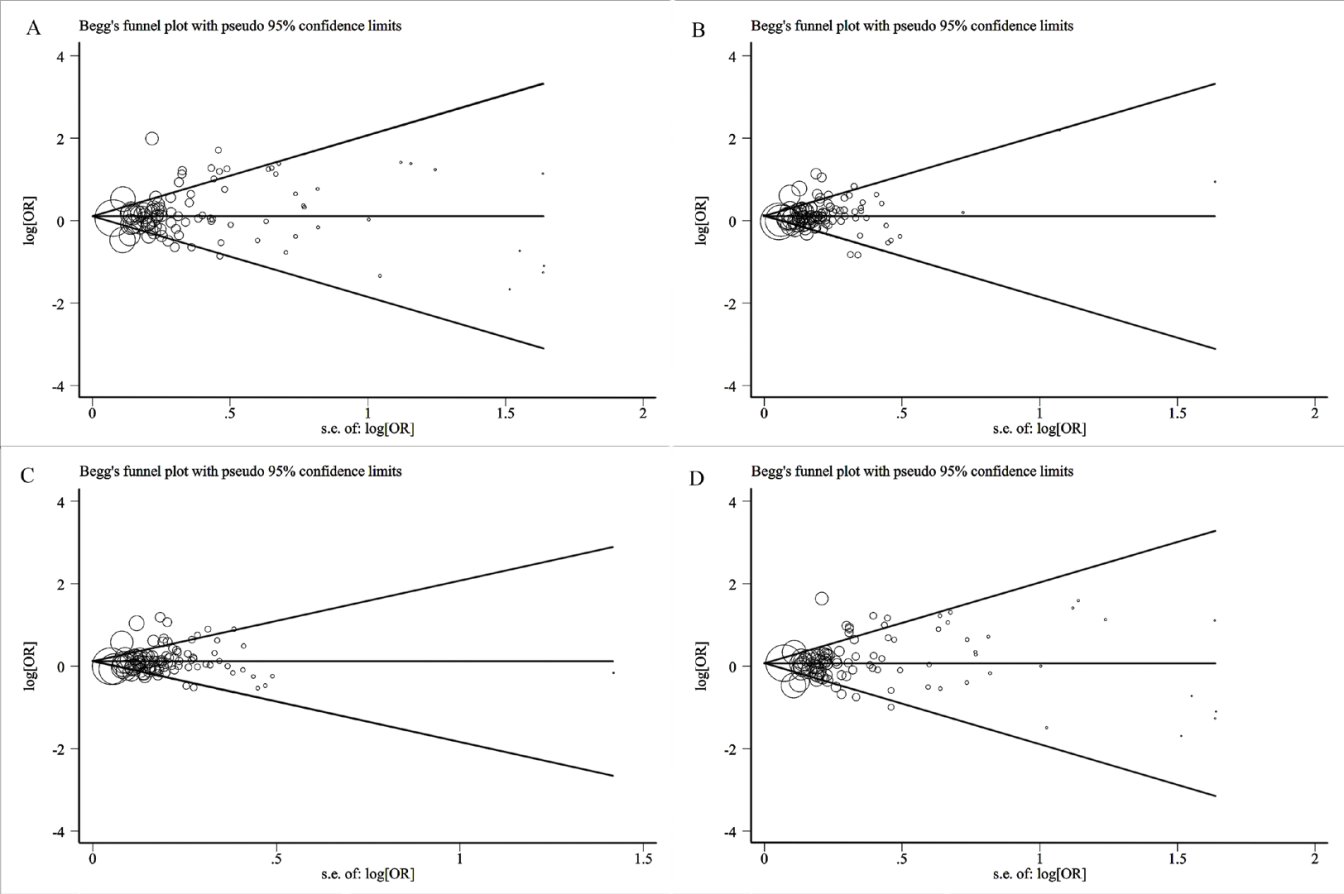
**(A)** Begg's funnel plot for the publication bias test in the overall analysis under homozygote comparison. **(B)** Begg's funnel plot for the publication bias test in the overall analysis under heterozygote comparison. **(C)** Begg's funnel plot for the publication bias test in the overall analysis under dominant model. **(D)** Begg's funnel plot for the publication bias test in the overall analysis under recessive model.

We used the Begg's funnel plot to estimate publication bias. There was no statistical evidence of publication bias in the overall analysis under each model (Figure 2). Table 2 shows the P details for bias. We also removed studies one by one to determine their effect on the test of heterogeneity, and evaluated the stability of the overall results; the results did not change in the overall analysis (Supplementary Table 1) neither in other analysis.

### Trial sequential analysis (TSA)

**Figure 3:**
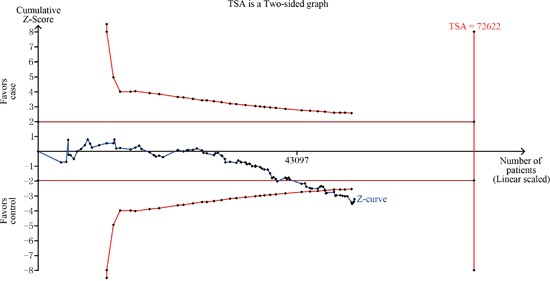
TSA for overall analysis under homozygote comparison

In the overall analysis for homozygote comparison, the required information size was 72,622 patients to demonstrate the issue (Figure 3), and the result showed that the Z-curve had crossed the trial monitoring boundary before reaching the required information size, indicating that the cumulative evidence is adequate and further trials are unnecessary.

## DISCUSSION

Nowadays, cancer is one of the most important global public health problems [106]. Personalized analysis and improved methods of cancer diagnoses can be provided, based on an understanding of the association between genetic polymorphisms and cancer risk [107]. In the relationship between gene polymorphisms and cancer risk, the *ERCC2* Asp312Asn polymorphism is an important risk factor. Impaired DNA repair capacity is a risk factor for the development of cancer. The *ERCC2* Asp312Asn polymorphism influences DNA repair through the NER pathway. To date, many publications have shown an association between the *ERCC2* Asp312Asn polymorphism and risk of cancer. However, the results remain controversial. In order to resolve this conflict, we performed a meta-analysis that evaluates the relationship between the *ERCC2* Asp312Asn polymorphism and risk of cancer.

In our meta-analysis, the association of the *ERCC2* Asp312Asn polymorphism with the risk of cancer was evaluated in 38,848 cases and 48,928 controls. A significant association was observed between the *ERCC2* Asp312Asn polymorphism and overall cancer risk in all genetic models. To the best of our knowledge, this is the most comprehensive meta-analysis on this topic until now. Moreover, the result of the TSA indicated that the cumulative evidence is adequate and further trials are unnecessary in the overall analysis for homozygote comparison.

In the subgroup analysis based on ethnicity, a significantly increased cancer risk was observed in Asian populations, but not in Caucasian populations. One possible reason for these discrepancies is that different ethnicities may have distinct genetic backgrounds, and therefore, tumor susceptibility can be influenced by ethnicity [108]. Moreover, this may indicate that these groups have distinct environmental or genetic cancer co-etiologies [109]. In subgroup analysis based on the control source, we found that a significantly increased cancer risk was observed in HB studies, but not in PB studies. The former may have certain biases for such controls and may only represent a sample of an ill-defined reference population. Furthermore, HB controls may not be representative of the general population or it may be that numerous subjects in the PB controls were individuals susceptible to cancer [110]. In the subgroup analysis based on the genotyping method, a significantly increased cancer risk was found in the PCR-RFLP studies, but not in the PCR or TaqMan studies. A possible reason for this may be that the different genotyping methods are specialized for different aspects, and the results would be more accurate and reliable if the same genotyping method was applied in different studies [111].

In the subgroup analysis according to the cancer site, a significant association with the *ERCC2* Asp312Asn polymorphism was observed for bladder, esophageal, and gastric cancers; however, no significant association was observed for breast, head and neck, lung, prostate, and skin cancers, and non- Hodgkin lymphoma. Some previous meta-analyses assessed the effect of the *ERCC2* Asp312Asn polymorphism on the risk of these cancers and reached conclusions consistent with those of our study. For example, Li et al. [19] and Wen et al. [14] suggested that the *ERCC2* Asp312Asn polymorphism might be associated with an increased risk of bladder cancer and esophageal cancer, respectively. Yin et al. [48] showed that this polymorphism might be a potential biomarker of gastric cancer susceptibility in the overall population. In contrast, Yan et al. [21], Hu et al. [11], and Zhu et al. [112] suggested that the *ERCC2* Asp312Asn polymorphism was not associated with breast cancer, head and neck cancer, and skin cancer, respectively. Moreover, Chen et al. [113], Feng et al. [12], and Ma et al. [114] suggested that the *ERCC2* Asp312Asn polymorphism contributed to the risk of non-Hodgkin lymphoma, lung cancer, and prostate cancer, respectively. Because we only included studies published from 2005 to 2016, we drew different conclusions in lung cancer and prostate cancer studies. Therefore, more research should be undertaken in the future. Moreover, the exact mechanism for the associations between different cancer sites and the *ERCC2* Asp312Asn polymorphism is not clear; the mechanism of carcinogenesis may differ between different cancer sites and the *ERCC2* genetic variants may exert varying effects in different cancers [115].

Notably, HCC, osteosarcoma, oral cancer, and colorectal cancer were not included for further analysis as there were fewer than 6 studies available for analysis for such cancers. Wu et al. indicated that the *ERCC2* Asp312Asn polymorphism was not associated with the development of HCC [24]. Gomez-Diaz et al. demonstrated no relationship between *ERCC2* Asp312Asn polymorphism and osteosarcoma [23]. Interestingly, based on a study by Mahimkar et al. this polymorphism was associated with an overall increase in chromosomal damage in oral cancer [25]. Wang et al. [35] observed a slightly lower statistical significance between the *ERCC2* Asp312Asn polymorphism and colorectal cancer. In fact, this polymorphism has also been shown to be related to other diseases; previous studies have indicated that it may have a role in the development of ultraviolet-related diseases, such as maturity onset cataract. [116]. However, no significant association of this polymorphism was found with either idiopathic azoospermia [117] or arsenic-related skin lesions [118]. Therefore, the equivocal association between the *ERCC2* Asp312Asn polymorphism and some diseases remains to be confirmed.

Heterogeneity is a major concern for meta-analysis [119]. In our overall analysis, high heterogeneity was observed for all genetic models. However, when data were pooled in to subgroups according the control source, ethnicity, genotyping method, and cancer type, the heterogeneity decreased. Sensitivity analysis showed that the results have sufficient statistical power. There are some limitations of our meta-analysis that should be addressed. First, subgroup analysis cannot be conducted based on sex, age, lifestyle, and other factors owing to insufficient data. Second, some cancers, such as oral cancer and colorectal cancer, were not suitable for further analysis because of the small sample sizes. Thus, more studies on these cancers should be conducted in the future. Third, a single gene has only a moderate effect on cancer development; hence, the *ERCC2* gene may influence susceptibility of cancer along with other genes. However, enough data for further analysis is not available. Finally, only published articles were included in the analysis; therefore, unpublished data may modify our conclusions.

In summary, our meta-analysis suggested that the *ERCC2* Asp312Asn polymorphism is associated with increased cancer risk. A significantly increased cancer risk was observed in Asian populations, but not in Caucasian populations. Moreover, our results indicated that this polymorphism is associated with bladder, esophageal, and gastric cancers, but not with breast, head and neck, lung, prostate, and skin cancers, and non-Hodgkin lymphoma. In addition, stratification analyses based on the control source also indicated that this polymorphism was associated with cancer risk in the HB populations, but not in the PB populations. In subgroup analysis according to the genotyping method, a significantly increased cancer risk was found in the PCR-RFLP studies, but not in the PCR and TaqMan studies. Considering the limitations of this study, further multi-center, well-designed research should be undertaken in the future.

## MATERIALS AND METHODS

### Literature search

A systematic search of articles relating to the *ERCC2* Asp312Asn polymorphism and cancer was conducted by 2 researchers, using the PubMed, EMBASE, Science Direct, Web of Science and the China National Knowledge Infrastructure (CNKI) databases. The search included studies published between January 1, 2005 and January 1, 2016. The search strategy was based on various combinations of the following terms: “xeroderma pigmentosum group d protein “[MeSH Terms] OR “xeroderma pigmentosum group d protein” [All Fields] OR “ercc2” [All Fields]) AND Asp312Asn [All Fields] AND (“neoplasms” [MeSH Terms] OR “neoplasms” [All Fields] OR “cancer” [All Fields]. In addition, the reference lists of the publications identified were searched for further relevant studies. The PRISMA Checklist was used for this meta-analysis (Supplementary Table 2).

### Selection criteria

The following inclusion criteria were set and reviewed by two independent investigators: (I) case-control study; (II) evaluation of the *ERCC2* Asp312Asn polymorphism and cancer; and (III) detailed data available for calculating ORs and the corresponding 95% CIs. Studies were excluded if they: (I) had no control population; (II) were review articles or previous meta-analyses; (III) contained insufficient or duplicate data; or (IV) had no full text available.

### Data extraction

Two authors performed data extraction independently. For all publications, the following data were extracted: first author, year of publication, ethnicity of the population, country, source of cases and controls, cancer site, genotyping method, and number of cases and controls.

### Trial sequential analysis

To evaluate whether our meta-analysis had sufficient sample size to reach firm conclusions about the effect of interventions [120], TSA was used in this meta-analysis. If the cumulative Z curve in results exceeds the TSA boundary, a sufficient level of evidence for the anticipated intervention effect may have been reached and no further trials are needed. However, when the Z curve does not exceed the TSA boundaries and the required information size has not been reached, evidence to draw a conclusion is insufficient [121]. We used two-sided tests, type I error set at 5%, and power set at 80%. The required information size was calculated based on a relative risk reduction of 10%. Trials ignored in interim appear to be due to too low use of information (<1.0%) by the software. TSA was performed using the TSA software (version 0.9.5.5).

### Statistical analysis

The primary objective of our meta-analysis was to calculate ORs and their 95% CIs to evaluate the association between *ERCC2* Asp312Asn and cancer risks. In our included studies, no clear models had been chosen; thus, the following genetic models were used: homozygote comparison (Asn/Asn vs. Asp/Asp), heterozygote comparison (Asp/Asn vs. Asp/Asp), recessive model (Asn/Asn vs. Asp/Asp+Asp/Asn), and dominant model (Asn/Asn+Asp/Asn vs. Asp/Asp). The statistical heterogeneity assumption was evaluated using I^2^ statistics to quantify any inconsistency arising from inter-research variability that was derived from heterogeneity instead of random chance [107]. An I^2^ value from 0-25% indicates low heterogeneity, 25-50% moderate heterogeneity and ≥50% high heterogeneity [122]. Two models (fixed-effect model and random-effect model) were used for analysis [123]. When I^2^< 50%, we used a fixed effect model and when I^2^ ≥50%, we performed a random effect model [124, 125]. We used sensitivity analyses by omitting each study in turn to determine the effect of heterogeneity on the test, and evaluated the stability of the overall results [107]. Potential publication bias was assessed using the Begg's linear regression test [126]. Notably, subgroup analysis was not performed when there were fewer than 6 studies available, because the small number may have resulted in insufficient power [107]. All statistical analyses were performed using the STATA statistical software package (version 12.0; StataCorp, College Station, TX).

## SUPPLEMENTARY MATERIALS TABLES







## Abbreviations

NER: nucleotide excision repair
ERCC2: excision repair cross-complementing group 2
XPD: Xeroderma pigmentosum group D
TFIIH: transcription factor IIH
SNPs: single nucleotide polymorphisms
Asn: asparagine amino acid
HB: hospital-based
PB: population-based
HCC: hepatocellular cancer
CNKI: China National Knowledge Infrastructure
TSA: trial sequential analysis

## References

[1] Hanahan D, Weinberg RA (2011). Hallmarks of cancer: the next generation. Cell.

[2] Castro J, Ribo M, Benito A, Vilanova M (2013). Mini-review: nucleus-targeted ribonucleases as antitumor drugs. Curr Med Chem.

[3] Siegel RL, Miller KD, Jemal A (2015). Cancer statistics, 2015. CA Cancer J Clin.

[4] Huang X, Gao Y, He J, Cai J, Ta N, Jiang H, Zhu J, Zheng J (2016). The association between RFC1 G80A polymorphism and cancer susceptibility: Evidence from 33 studies. J Cancer.

[5] Qixing M, Gaochao D, Wenjie X, Rong Y, Feng J, Lin X, Mantang Q, Qiang C (2015). Predictive Value of Ercc1 and Xpd Polymorphisms for Clinical Outcomes of Patients Receiving Neoadjuvant Therapy: A Prisma-Compliant Meta-Analysis. Medicine (Baltimore).

[6] Benhamou S, Sarasin A (2005). ERCC2 /XPD gene polymorphisms and lung cancer: a HuGE review. Am J Epidemiol.

[7] Alanazi M, Pathan AA, Ajaj SA, Khan W, Shaik JP, Al Tassan N, Parine NR (2013). DNA Repair Genes XRCC1, XRCC3, XPD, and OGG1 Polymorphisms among the Central Region Population of Saudi Arabia. Biol Res.

[8] Ouyang FD, Yang FL, Chen HC, Khan MA, Huang FM, Wan XX, Xu AH, Huang X, Zhou MJ, Fang Q, Zhang DZ (2013). Polymorphisms of DNA repair genes XPD, XRCC1, and OGG1, and lung adenocarcinoma susceptibility in Chinese population. Tumour Biol.

[9] Clarkson SG, Wood RD (2005). Polymorphisms in the human XPD (ERCC2) gene, DNA repair capacity and cancer susceptibility: an appraisal. DNA Repair (Amst).

[10] Lunn RM, Helzlsouer KJ, Parshad R, Umbach DM, Harris EL, Sanford KK, Bell DA (2000). XPD polymorphisms: effects on DNA repair proficiency. Carcinogenesis.

[11] Hu YY, Yuan H, Jiang GB, Chen N, Wen L, Leng WD, Zeng XT, Niu YM (2012). Associations between XPD Asp312Asn polymorphism and risk of head and neck cancer: a meta-analysis based on 7,122 subjects. PLoS One.

[12] Feng Z, Ni Y, Dong W, Shen H, Du J (2012). Association of ERCC2/XPD polymorphisms and interaction with tobacco smoking in lung cancer susceptibility: a systemic review and meta-analysis. Mol Biol Rep.

[13] Geng P, Ou J, Li J, Liao Y, Wang N, Xie G, Sa R, Liu C, Xiang L, Liang H (2015). A Comprehensive Analysis of Influence ERCC Polymorphisms Confer on the Development of Brain Tumors. Mol Neurobiol.

[14] Wen F, Zhao Z, Liu C, Yin Q, Weng J, Wang Y, Ma Y (2014). A pooled analysis of the ERCC2 Asp312Asn polymorphism and esophageal cancer susceptibility. Tumour Biol.

[15] Zhu ML, He J, Wang M, Sun MH, Jin L, Wang X, Yang YJ, Wang JC, Zheng L, Xiang JQ, Wei QY (2014). Potentially functional polymorphisms in the ERCC2 gene and risk of esophageal squamous cell carcinoma in Chinese populations. Sci Rep.

[16] Yang R, Zhang C, Malik A, Shen ZD, Hu J, Wu YH (2014). Xeroderma pigmentosum group D polymorphisms and esophageal cancer susceptibility: a meta-analysis based on case-control studies. World J Gastroenterol.

[17] Li C, Jiang Z, Liu X (2010). XPD Lys (751) Gln and Asp (312) Asn polymorphisms and bladder cancer risk: a meta-analysis. Mol Biol Rep.

[18] Wu Y, Yang Y (2014). Complex association between ERCC2 gene polymorphisms, gender, smoking and the susceptibility to bladder cancer: a meta-analysis. Tumour Biol.

[19] Li SX, Dai QS, Chen SX, Zhang SD, Liao XY, Deng X, Chi HB, Li FJ, Zhu JH, Jiang YY (2014). Xeroderma pigmentosum complementation group D (XPD) gene polymorphisms contribute to bladder cancer risk: a meta-analysis. Tumour Biol.

[20] Jiang Z, Li C, Xu Y, Cai S, Wang X (2010). Associations between XPD polymorphisms and risk of breast cancer: a meta-analysis. Breast Cancer Res Treat.

[21] Yan Y, Liang H, Light M, Li T, Deng Y, Li M, Li S, Qin X (2014). XPD Asp312Asn and Lys751Gln polymorphisms and breast cancer susceptibility: a meta-analysis. Tumour Biol.

[22] Yao L, Qiu LX, Yu L, Yang Z, Yu XJ, Zhong Y, Hu XC, Yu L (2010). The association between ERCC2 Asp312Asn polymorphism and breast cancer risk: a meta-analysis involving 22,766 subjects. Breast Cancer Res Treat.

[23] Gómez-Díaz B, de la Luz Ayala-Madrigal M, Gutiérrez-Angulo M, Valle-Solis AE, Linares-González LM, González-Guzmán R, Cruz-Guillén D, Cedeño-Garcidueñas AL, Canto P, López-Hernández LB (2015). Analysis of ERCC1 and ERCC2 gene variants in osteosarcoma, colorectal and breast cancer. Oncol Lett.

[24] Wu JS, Chen YP, Wang LC, Yang YJ, Deng CW, Hou BX, He ZL, Chen JX (2014). Implication of polymorphisms in DNA repair genes with an increased risk of hepatocellular carcinoma. Genet Mol Res.

[25] Mahimkar MB, Samant TA, Kannan S, Patil T (2010). Influence of genetic polymorphisms on frequency of micronucleated buccal epithelial cells in leukoplakia patients. Oral Oncol.

[26] Wang Y, Spitz MR, Lee JJ, Huang M, Lippman SM, Wu X (2007). Nucleotide excision repair pathway genes and oral premalignant lesions. Clin Cancer Res.

[27] Majumder M, Sikdar N, Ghosh S, Roy B (2007). Polymorphisms at XPD and XRCC1 DNA repair loci and increased risk of oral leukoplakia and cancer among NAT2 slow acetylators. Int J Cancer.

[28] Han J, Colditz GA, Liu JS, Hunter DJ (2005). Genetic variation in XPD, sun exposure, and risk of skin cancer. Cancer Epidemiol Biomarkers Prev.

[29] Lovatt T, Alldersea J, Lear JT, Hoban PR, Ramachandran S, Fryer AA, Smith AG, Strange RC (2005). Polymorphism in the nuclear excision repair gene ERCC2/XPD: association between an exon 6-exon 10 haplotype and susceptibility to cutaneous basal cell carcinoma. Hum Mutat.

[30] Li C, Hu Z, Liu Z, Wang LE, Strom SS, Gershenwald JE, Lee JE, Ross MI, Mansfield PF, Cormier JN, Prieto VG, Duvic M, Grimm EA (2006). Polymorphisms in the DNA repair genes XPC, XPD, and XPG and risk of cutaneous melanoma: a case-control analysis. Cancer Epidemiol Biomarkers Prev.

[31] Millikan RC, Hummer A, Begg C, Player J, de Cotret AR, Winkel S, Mohrenweiser H, Thomas N, Armstrong B, Kricker A, Marrett LD, Gruber SB, Culver HA (2006). Polymorphisms in nucleotide excision repair genes and risk of multiple primary melanoma: the Genes Environment and Melanoma Study. Carcinogenesis.

[32] Debniak T, Scott RJ, Huzarski T, Byrski T, Masojc B, van de Wetering T, Serrano-Fernandez P, Gorski B, Cybulski C, Gronwald J, Debniak B, Maleszka R, Kladny J (2006). XPD common variants and their association with melanoma and breast cancer risk. Breast Cancer Res Treat.

[33] Moreno V, Gemignani F, Landi S, Gioia-Patricola L, Chabrier A, Blanco I, Gonzalez S, Guino E, Capella G, Canzian F (2006). Polymorphisms in genes of nucleotide and base excision repair: risk and prognosis of colorectal cancer. Clin Cancer Res.

[34] Hansen RD, Sorensen M, Tjonneland A, Overvad K, Wallin H, Raaschou-Nielsen O, Vogel U (2007). XPA A23G, XPC Lys939Gln, XPD Lys751Gln and XPD Asp312Asn polymorphisms, interactions with smoking, alcohol and dietary factors, and risk of colorectal cancer. Mutat Res.

[35] Joshi AD, Corral R, Siegmund KD, Haile RW, Le Marchand L, Martinez ME, Ahnen DJ, Sandler RS, Lance P, Stern MC (2009). Red meat and poultry intake, polymorphisms in the nucleotide excision repair and mismatch repair pathways and colorectal cancer risk. Carcinogenesis.

[36] Ni M, Zhang WZ, Qiu JR, Liu F, Li M, Zhang YJ, Liu Q, Bai J (2014). Association of ERCC1 and ERCC2 polymorphisms with colorectal cancer risk in a Chinese population. Sci Rep.

[37] An J, Liu Z, Hu Z, Li G, Wang LE, Sturgis EM, El-Naggar AK, Spitz MR, Wei Q (2007). Potentially functional single nucleotide polymorphisms in the core nucleotide excision repair genes and risk of squamous cell carcinoma of the head and neck. Cancer Epidemiol Biomarkers Prev.

[38] Harth V, Schafer M, Abel J, Maintz L, Neuhaus T, Besuden M, Primke R, Wilkesmann A, Thier R, Vetter H, Ko YD, Bruning T, Bolt HM (2008). Head and neck squamous-cell cancer and its association with polymorphic enzymes of xenobiotic metabolism and repair. J Toxicol Environ Health A.

[39] Abbasi R, Ramroth H, Becher H, Dietz A, Schmezer P, Popanda O (2009). Laryngeal cancer risk associated with smoking and alcohol consumption is modified by genetic polymorphisms in ERCC5, ERCC6 and RAD23B but not by polymorphisms in five other nucleotide excision repair genes. Int J Cancer.

[40] Ji YB, Tae K, Lee YS, Lee SH, Kim KR, Park CW, Park BL, Shin HD (2010). XPD Polymorphisms and Risk of Squamous Cell Carcinoma of the Head and Neck in a Korean Sample. Clin Exp Otorhinolaryngol.

[41] Gugatschka M, Dehchamani D, Wascher TC, Friedrich G, Renner W (2011). DNA repair gene ERCC2 polymorphisms and risk of squamous cell carcinoma of the head and neck. Exp Mol Pathol.

[42] Das S, Bhowmik A, Bhattacharjee A, Choudhury B, Naiding M, Laskar AK, Ghosh SK, Choudhury Y (2015). XPD, APE1, and MUTYH polymorphisms increase head and neck cancer risk: effect of gene-gene and gene-environment interactions. Tumour Biol.

[43] Liu G, Zhou W, Yeap BY, Su L, Wain JC, Poneros JM, Nishioka NS, Lynch TJ, Christiani DC (2007). XRCC1 and XPD polymorphisms and esophageal adenocarcinoma risk. Carcinogenesis.

[44] Ye W, Kumar R, Bacova G, Lagergren J, Hemminki K, Nyren O (2006). The XPD 751Gln allele is associated with an increased risk for esophageal adenocarcinoma: a population-based case-control study in Sweden. Carcinogenesis.

[45] Tse D, Zhai R, Zhou W, Heist RS, Asomaning K, Su L, Lynch TJ, Wain JC, Christiani DC, Liu G (2008). Polymorphisms of the NER pathway genes, ERCC1 and XPD are associated with esophageal adenocarcinoma risk. Cancer Causes Control.

[46] Pan J, Lin J, Izzo JG, Liu Y, Xing J, Huang M, Ajani JA, Wu X (2009). Genetic susceptibility to esophageal cancer: the role of the nucleotide excision repair pathway. Carcinogenesis.

[47] Huang CG, Liu T, Lv GD, Liu Q, Feng JG, Lu XM (2012). Analysis of XPD genetic polymorphisms of esophageal squamous cell carcinoma in a population of Yili Prefecture, in Xinjiang, China. Mol Biol Rep.

[48] Yin QH, Liu C, Hu JB, Meng RR, Li L, Wang YJ (2013). XPD Lys751Gln and Asp312Asn polymorphisms and gastric cancer susceptibility: a meta-analysis of case-control studies. Asian Pac J Cancer Prev.

[49] Smedby KE, Lindgren CM, Hjalgrim H, Humphreys K, Schollkopf C, Chang ET, Roos G, Ryder LP, Falk KI, Palmgren J, Kere J, Melbye M, Glimelius B (2006). Variation in DNA repair genes ERCC2, XRCC1, and XRCC3 and risk of follicular lymphoma. Cancer Epidemiol Biomarkers Prev.

[50] Shen M, Zheng T, Lan Q, Zhang Y, Zahm SH, Wang SS, Holford TR, Leaderer B, Yeager M, Welch R, Kang D, Boyle P, Zhang B (2006). Polymorphisms in DNA repair genes and risk of non-Hodgkin lymphoma among women in Connecticut. Hum Genet.

[51] Song B, Zhu JY, Liu J, Wang ZH, Shi Y, Lü LY, Zheng Y (2008). [Association of Gene Polymorphisms in the DNA Repair Gene XPD with Risk of Non-Hodgkin's Lymphoma]. [Article in Chinese]. Zhongguo Shi Yan Xue Ye Xue Za Zhi.

[52] Baris S, Celkan T, Batar B, Guven M, Ozdil M, Ozkan A, Apak H, Yildiz I (2009). Association between genetic polymorphism in DNA repair genes and risk of B-cell lymphoma. Pediatr Hematol Oncol.

[53] Worrillow L, Roman E, Adamson PJ, Kane E, Allan JM, Lightfoot TJ (2009). Polymorphisms in the nucleotide excision repair gene ERCC2/XPD and risk of non-Hodgkin lymphoma. Cancer Epidemiol.

[54] El-Din MA, Khorshied MM, El-Saadany ZA, El-Banna MA, Reda Khorshid OM (2013). Excision repair cross-complementing group 2/Xeroderma pigmentousm complementation group D (ERCC2/XPD) genetic variations and susceptibility to diffuse large B cell lymphoma in Egypt. Int J Hematol.

[55] Agalliu I, Kwon EM, Salinas CA, Koopmeiners JS, Ostrander EA, Stanford JL (2010). Genetic variation in DNA repair genes and prostate cancer risk: results from a population-based study. Cancer Causes Control.

[56] Mirecka A, Paszkowska-Szczur K, Scott RJ, Gorski B, van de Wetering T, Wokolorczyk D, Gromowski T, Serrano-Fernandez P, Cybulski C, Kashyap A, Gupta S, Golab A, Slojewski M (2014). Common variants of xeroderma pigmentosum genes and prostate cancer risk. Gene.

[57] Bau DT, Wu HC, Chiu CF, Lin CC, Hsu CM, Wang CL, Wang RF, Tsai FJ (2007). Association of XPD polymorphisms with prostate cancer in Taiwanese patients. Anticancer Res.

[58] Mandal RK, Gangwar R, Mandhani A, Mittal RD (2010). DNA repair gene X-ray repair cross-complementing group 1 and xeroderma pigmentosum group D polymorphisms and risk of prostate cancer: a study from North India. DNA Cell Biol.

[59] Lavender NA, Komolafe OO, Benford M, Brock G, Moore JH, Vancleave TT, States JC, Kittles RA, Kidd LC (2010). No association between variant DNA repair genes and prostate cancer risk among men of African descent. Prostate.

[60] Dhillon VS, Yeoh E, Fenech M (2011). DNA repair gene polymorphisms and prostate cancer risk in South Australia—results of a pilot study. Urol Oncol.

[61] Yuan T, Deng S, Chen M, Chen W, Lu W, Huang H, Xia J (2011). Association of DNA repair gene XRCC1 and XPD polymorphisms with genetic susceptibility to gastric cancer in a Chinese population. Cancer Epidemiol.

[62] Chen Z, Zhang C, Xu C, Li K, Hou R, Li D, Cheng X (2011). Effects of selected genetic polymorphisms in xeroderma pigmentosum complementary group D on gastric cancer. Mol Biol Rep.

[63] Ruzzo A, Canestrari E, Maltese P, Pizzagalli F, Graziano F, Santini D, Catalano V, Ficarelli R, Mari D, Bisonni R, Giordani P, Giustini L, Lippe P (2007). Polymorphisms in genes involved in DNA repair and metabolism of xenobiotics in individual susceptibility to sporadic diffuse gastric cancer. Clin Chem Lab Med.

[64] Yuan T, Deng S, Chen M, Chen W, Lu W, Huang H, Xia J (2010). Association of DNA repair gene polymorphisms with genetic susceptibility to gastric cancer. J Mol Diagn Ther.

[65] Zhang CZ, Chen ZP, Xu CQ, Ning T, Li DP, Hou RP (2009). [Correlation of XPD gene with susceptibility to gastric cancer]. [Article in Chinese]. Ai Zheng.

[66] Yi L, Song BQ, He XM (2006). [Association of single nucleotide polymorphism in DNA repair gene XPD with gastric cancer in Han population from northeast region of China]. [Article in Chinese]. Shijie Huaren Xiaohua Zazhi.

[67] Zhou R, Li Y, Wand N, Dong X, Zhang X, Guo W (2007). Correlation between single nucleotide polymorphism of DNA repair gene XPD and the risks of esophageal squamous cell carcinoma and gastric cardiacadeno carcinoma. Epidemiological Research.

[68] Broberg K, Bjork J, Paulsson K, Hoglund M, Albin M (2005). Constitutional short telomeres are strong genetic susceptibility markers for bladder cancer. Carcinogenesis.

[69] Matullo G, Guarrera S, Sacerdote C, Polidoro S, Davico L, Gamberini S, Karagas M, Casetta G, Rolle L, Piazza A, Vineis P (2005). Polymorphisms/haplotypes in DNA repair genes and smoking: a bladder cancer case-control study. Cancer Epidemiol Biomarkers Prev.

[70] Matullo G, Dunning AM, Guarrera S, Baynes C, Polidoro S, Garte S, Autrup H, Malaveille C, Peluso M, Airoldi L, Veglia F, Gormally E, Hoek G (2006). DNA repair polymorphisms and cancer risk in non-smokers in a cohort study. Carcinogenesis.

[71] Schabath MB, Delclos GL, Grossman HB, Wang Y, Lerner SP, Chamberlain RM, Spitz MR, Wu X (2005). Polymorphisms in XPD exons 10 and 23 and bladder cancer risk. Cancer Epidemiol Biomarkers Prev.

[72] Andrew AS, Nelson HH, Kelsey KT, Moore JH, Meng AC, Casella DP, Tosteson TD, Schned AR, Karagas MR (2006). Concordance of multiple analytical approaches demonstrates a complex relationship between DNA repair gene SNPs, smoking and bladder cancer susceptibility. Carcinogenesis.

[73] Garcia-Closas M, Malats N, Real FX, Welch R, Kogevinas M, Chatterjee N, Pfeiffer R, Silverman D, Dosemeci M, Tardon A, Serra C, Carrato A, Garcia-Closas R (2006). Genetic variation in the nucleotide excision repair pathway and bladder cancer risk. Cancer Epidemiol Biomarkers Prev.

[74] Wu X, Gu J, Grossman HB, Amos CI, Etzel C, Huang M, Zhang Q, Millikan RE, Lerner S, Dinney CP, Spitz MR (2006). Bladder cancer predisposition: a multigenic approach to DNA-repair and cell-cycle-control genes. Am J Hum Genet.

[75] Fontana L, Bosviel R, Delort L, Guy L, Chalabi N, Kwiatkowski F, Satih S, Rabiau N, Boiteux JP, Chamoux A, Bignon YJ, Bernard-Gallon DJ (2008). DNA repair gene ERCC2, XPC, XRCC1, XRCC3 polymorphisms and associations with bladder cancer risk in a French cohort. Anticancer Res.

[76] Chang CH, Wang RF, Tsai RY, Wu HC, Wang CH, Tsai CW, Chang CL, Tsou YA, Liu CS, Bau DT (2009). Significant association of XPD codon 312 single nucleotide polymorphism with bladder cancer susceptibility in Taiwan. Anticancer Res.

[77] Gangwar R, Ahirwar D, Mandhani A, Mittal RD (2009). Influence of XPD and APE1 DNA repair gene polymorphism on bladder cancer susceptibility in north India. Urology.

[78] Mittal RD, Mandal RK (2012). Genetic variation in nucleotide excision repair pathway genes influence prostate and bladder cancer susceptibility in North Indian population. Indian J Hum Genet.

[79] Ramaniuk VP, Nikitchenko NV, Savina NV, Kuzhir TD, Rolevich AI, Krasny SA, Sushinsky VE, Goncharova RI (2014). Polymorphism of DNA repair genes OGG1, XRCC1, XPD and ERCC6 in bladder cancer in Belarus. Biomarkers.

[80] Zhou M, Wan HY, Gao BL, Ding YJ, Jun RX (2012). Genetic polymorphisms of XPD and CDA and lung cancer risk. Oncol Lett.

[81] Sakoda LC, Loomis MM, Doherty JA, Julianto L, Barnett MJ, Neuhouser ML, Thornquist MD, Weiss NS, Goodman GE, Chen C (2012). Germ line variation in nucleotide excision repair genes and lung cancer risk in smokers. Int J Mol Epidemiol Genet.

[82] Qian B, Zhang H, Zhang L, Zhou X, Yu H, Chen K (2011). Association of genetic polymorphisms in DNA repair pathway genes with non-small cell lung cancer risk. Lung Cancer.

[83] Yin Z, Su M, Li X, Li M, Ma R, He Q, Zhou B (2009). ERCC2, ERCC1 polymorphisms and haplotypes, cooking oil fume and lung adenocarcinoma risk in Chinese non-smoking females. J Exp Clin Cancer Res.

[84] Raaschou-Nielsen O, Sorensen M, Overvad K, Tjonneland A, Vogel U (2008). Polymorphisms in nucleotide excision repair genes, smoking and intake of fruit and vegetables in relation to lung cancer. Lung Cancer.

[85] Chang JS, Wrensch MR, Hansen HM, Sison JD, Aldrich MC, Quesenberry CP, Seldin MF, Kelsey KT, Kittles RA, Silva G, Wiencke JK (2008). Nucleotide excision repair genes and risk of lung cancer among San Francisco Bay Area Latinos and African Americans. Int J Cancer.

[86] Yin J, Vogel U, Ma Y, Qi R, Sun Z, Wang H (2007). A haplotype encompassing the variant allele of DNA repair gene polymorphism ERCC2/XPD Lys751Gln but not the variant allele of Asp312Asn is associated with risk of lung cancer in a northeastern Chinese population. Cancer Genet Cytogenet.

[87] Lopez-Cima MF, Gonzalez-Arriaga P, Garcia-Castro L, Pascual T, Marron MG, Puente XS, Tardon A (2007). Polymorphisms in XPC, XPD, XRCC1, and XRCC3 DNA repair genes and lung cancer risk in a population of northern Spain. BMC Cancer.

[88] Zienolddiny S, Campa D, Lind H, Ryberg D, Skaug V, Stangeland L, Phillips DH, Canzian F, Haugen A (2006). Polymorphisms of DNA repair genes and risk of non-small cell lung cancer. Carcinogenesis.

[89] Hu Z, Xu L, Shao M, Yuan J, Wang Y, Wang F, Yuan W, Qian J, Ma H, Wang Y, Liu H, Chen W, Yang L (2006). Polymorphisms in the two helicases ERCC2/XPD and ERCC3/XPB of the transcription factor IIH complex and risk of lung cancer: a case-control analysis in a Chinese population. Cancer Epidemiol Biomarkers Prev.

[90] Shen M, Berndt SI, Rothman N, Demarini DM, Mumford JL, He X, Bonner MR, Tian L, Yeager M, Welch R, Chanock S, Zheng T, Caporaso N (2005). Polymorphisms in the DNA nucleotide excision repair genes and lung cancer risk in Xuan Wei, China. Int J Cancer.

[91] Huang WY, Berndt SI, Kang D, Chatterjee N, Chanock SJ, Yeager M, Welch R, Bresalier RS, Weissfeld JL, Hayes RB (2006). Nucleotide excision repair gene polymorphisms and risk of advanced colorectal adenoma: XPC polymorphisms modify smoking-related risk. Cancer Epidemiol Biomarkers Prev.

[92] De Ruyck K, Szaumkessel M, De Rudder I, Dehoorne A, Vral A, Claes K, Velghe A, Van Meerbeeck J, Thierens H (2007). Polymorphisms in base-excision repair and nucleotide-excision repair genes in relation to lung cancer risk. Mutat Res.

[93] Crew KD, Gammon MD, Terry MB, Zhang FF, Zablotska LB, Agrawal M, Shen J, Long CM, Eng SM, Sagiv SK, Teitelbaum SL, Neugut AI, Santella RM (2007). Polymorphisms in nucleotide excision repair genes, polycyclic aromatic hydrocarbon-DNA adducts, and breast cancer risk. Cancer Epidemiol Biomarkers Prev.

[94] Kuschel B, Chenevix-Trench G, Spurdle AB, Chen X, Hopper JL, Giles GG, McCredie M, Chang-Claude J, Gregory CS, Day NE, Easton DF, Ponder BA, Dunning AM (2005). Common polymorphisms in ERCC2 (Xeroderma pigmentosum D) are not associated with breast cancer risk. Cancer Epidemiol Biomarkers Prev.

[95] Jorgensen TJ, Visvanathan K, Ruczinski I, Thuita L, Hoffman S, Helzlsouer KJ (2007). Breast cancer risk is not associated with polymorphic forms of xeroderma pigmentosum genes in a cohort of women from Washington County, Maryland. Breast Cancer Res Treat.

[96] Lee SA, Lee KM, Park WY, Kim B, Nam J, Yoo KY, Noh DY, Ahn SH, Hirvonen A, Kang D (2005). Obesity and genetic polymorphism of ERCC2 and ERCC4 as modifiers of risk of breast cancer. Exp Mol Med.

[97] Bernard-Gallon D, Bosviel R, Delort L, Fontana L, Chamoux A, Rabiau N, Kwiatkowski F, Chalabi N, Satih S, Bignon YJ (2008). DNA repair gene ERCC2 polymorphisms and associations with breast and ovarian cancer risk. Mol Cancer.

[98] Jakubowska A, Gronwald J, Menkiszak J, Gorski B, Huzarski T, Byrski T, Toloczko-Grabarek A, Gilbert M, Edler L, Zapatka M, Eils R, Lubinski J, Scott RJ (2010). BRCA1-associated breast and ovarian cancer risks in Poland: no association with commonly studied polymorphisms. Breast Cancer Res Treat.

[99] Mechanic LE, Millikan RC, Player J, de Cotret AR, Winkel S, Worley K, Heard K, Heard K, Tse CK, Keku T (2006). Polymorphisms in nucleotide excision repair genes, smoking and breast cancer in African Americans and whites: a population-based case-control study. Carcinogenesis.

[100] Shen J, Desai M, Agrawal M, Kennedy DO, Senie RT, Santella RM, Terry MB (2006). Polymorphisms in nucleotide excision repair genes and DNA repair capacity phenotype in sisters discordant for breast cancer. Cancer Epidemiol Biomarkers Prev.

[101] Smith TR, Levine EA, Freimanis RI, Akman SA, Allen GO, Hoang KN, Liu-Mares W, Hu JJ (2008). Polygenic model of DNA repair genetic polymorphisms in human breast cancer risk. Carcinogenesis.

[102] Zhang L, Zhang Z, Yan W (2005). Single nucleotide polymorphisms for DNA repair genes in breast cancer patients. Clin Chim Acta.

[103] Hussien YM, Gharib AF, Awad HA, Karam RA, Elsawy WH (2012). Impact of DNA repair genes polymorphism (XPD and XRCC1) on the risk of breast cancer in Egyptian female patients. Mol Biol Rep.

[104] Jelonek K, Gdowicz-Klosok A, Pietrowska M, Borkowska M, Korfanty J, Rzeszowska-Wolny J, Widlak P (2010). Association between single-nucleotide polymorphisms of selected genes involved in the response to DNA damage and risk of colon, head and neck, and breast cancers in a Polish population. J Appl Genet.

[105] Wang HC, Liu CS, Wang CH, Tsai RY, Tsai CW, Wang RF, Chang CH, Chen YS, Chiu CF, Bau DT, Huang CY (2010). Significant association of XPD Asp312Asn polymorphism with breast cancer in Taiwanese patients. Chin J Physiol.

[106] Lin QJ, Yang F, Jin C, Fu DL (2015). Current status and progress of pancreatic cancer in China. World J Gastroenterol.

[107] Zhang M, Wang Y, Fang T, Cai Y, Xu Y, Yan C, Zhang L, Liang C (2016). Common polymorphisms in CD44 gene and susceptibility to cancer: A systematic review and meta-analysis of 45 studies. Oncotarget.

[108] Zhang CD, Li HT, Liu K, Lin ZD, Peng QL, Qin X, He M, Wu H, Mo ZN, Yang XL (2014). Impact of caspase-8 (CASP8) -652 6N del and D302H polymorphisms on prostate cancer in different ethnic groups. Asian Pac J Cancer Prev.

[109] Fu W, Zhuo ZJ, Chen YC, Zhu J, Zhao Z, Jia W, Hu JH, Fu K, Zhu SB, He J, Liu GC (2016). NFKB1-94insertion/deletion ATTG polymorphism and cancer risk: Evidence from 50 case-control studies. Oncotarget.

[110] Xie SZ, Liu ZZ, Yu JH, Liu L, Wang W, Xie DL, Qin JB (2015). Association between the MTHFR C677T polymorphism and risk of cancer: evidence from 446 case-control studies. Tumour Biol.

[111] Tang J, Li X, Jiang X, Xu W, Xu Z, Wang W, Liu B, Lv Q, Zhang W (2015). The association between rs9642880 gene polymorphism and bladder cancer risk: a meta-analysis. Int J Clin Exp Med.

[112] Zhu HL, Bao JM, Lin PX, Li WX, Zou ZN, Huang YE, Chen Q, Shen H (2014). XPD Lys751Gln and Asp312Asn polymorphisms and susceptibility to skin cancer: a meta-analysis of 17 case-control studies. Asian Pac J Cancer Prev.

[113] Chen S, Zhu JH, Wang F, Huang SY, Xue WQ, Cui Z, He J, Jia WH (2015). Association of the Asp312Asn and Lys751Gln polymorphisms in the XPD gene with the risk of non-Hodgkin's lymphoma: evidence from a meta-analysis. Chin J Cancer.

[114] Ma Q, Qi C, Tie C, Guo Z (2013). Genetic polymorphisms of xeroderma pigmentosum group D gene Asp312Asn and Lys751Gln and susceptibility to prostate cancer: a systematic review and meta-analysis. Gene.

[115] Wu KG, He XF, Li YH, Xie WB, Huang X (2014). Association between the XPD/ERCC2 Lys751Gln polymorphism and risk of cancer: evidence from 224 case-control studies. Tumour Biol.

[116] Padma G, Mamata M, Reddy KR, Padma T (2011). Polymorphisms in two DNA repair genes (XPD and XRCC1)—association with age related cataracts. Mol Vis.

[117] Ji G, Gu A, Xia Y, Lu C, Liang J, Wang S, Ma J, Peng Y, Wang X (2008). ERCC1 and ERCC2 polymorphisms and risk of idiopathic azoospermia in a Chinese population. Reprod Biomed Online.

[118] McCarty KM, Smith TJ, Zhou W, Gonzalez E, Quamruzzaman Q, Rahman M, Mahiuddin G, Ryan L, Su L, Christiani DC (2007). Polymorphisms in XPD (Asp312Asn and Lys751Gln) genes, sunburn and arsenic-related skin lesions. Carcinogenesis.

[119] Xiao F, Lan A, Lin Z, Song J, Zhang Y, Li J, Gu K, Lv B, Zhao D, Zeng S, Zhang R, Zhao W, Pan Z (2016). Impact of CAG repeat length in the androgen receptor gene on male infertility - a meta-analysis. Reprod Biomed Online.

[120] Varvaki Rados D, Catani Pinto L, Reck Remonti L, Bauermann Leitao C, Gross JL (2016). The Association between Sulfonylurea Use and All-Cause and Cardiovascular Mortality: A Meta-Analysis with Trial Sequential Analysis of Randomized Clinical Trials. PLoS Med.

[121] Wang G, Zhang L, Lou S, Chen Y, Cao Y, Wang R, Zhang L, Tang P (2016). Effect of Dexmedetomidine in Preventing Postoperative Side Effects for Laparoscopic Surgery: A Meta-Analysis of Randomized Controlled Trials and Trial Sequential Analysis (PRISMA). Medicine (Baltimore).

[122] Zhang Y, Li M, Xiao F, Teng R, Zhang C, Lan A, Gu K, Li J, Wang D, Li H, Jiang L, Zeng S, He M (2015). Impact of partial DAZ1/2 deletion and partial DAZ3/4 deletion on male infertility. Gene.

[123] Higgins JP, Thompson SG, Deeks JJ, Altman DG (2003). Measuring inconsistency in meta-analyses. Bmj.

[124] Zhai Y, Dai Z, He H, Gao F, Yang L, Dong Y, Lu J (2016). A PRISMA-compliant meta-analysis of MDM4 genetic variants and cancer susceptibility. Oncotarget.

[125] Luo M, Yang Y, Luo D, Liu L, Zhang Y, Xiao F, Yang J, Zhang C, Fu S, Luo Z (2016). Tumor necrosis factor-alpha promoter polymorphism 308 G/A is not significantly associated with esophageal cancer risk: a meta-analysis. Oncotarget.

[126] Peters JL, Sutton AJ, Jones DR, Abrams KR, Rushton L (2006). Comparison of two methods to detect publication bias in meta-analysis. Jama.

