# Supplementary material for: Association between the ERCC2 Asp312Asn polymorphism and risk of cancer
Source: Oncotarget. 2017 Apr 20;8(29):48488–506. doi: 10.18632/oncotarget.17290 (PMC5564664; doi:10.18632/oncotarget.17290)
Supplement: Supplementary file 1 [file oncotarget-08-48488-s001.pdf]

Association between the *ERCC2* Asp312Asn polymorphism and risk of cancer

SUPPLEMENTARY TABLES

Supplementary Table 1: Results of sensitivity analysis in overall analysis under homozygote comparison

See Supplementary File 1

Supplementary Table 2: PRISMA Checklist

See Supplementary File 2
