# Supplementary material for: Association between the ERCC2 Asp312Asn polymorphism and risk of cancer
Source: Oncotarget. 2017 Apr 20;8(29):48488–506. doi: 10.18632/oncotarget.17290 (PMC5564664; doi:10.18632/oncotarget.17290)
Supplement: Supplementary file 2 [file oncotarget-08-48488-s002.docx]

**Supplementary table 1**: Results of sensitivity analysis in overall analysis under homozygote comparison

| Study ommited | Fixed model | | | Random model | | |
| --- | --- | --- | --- | --- | --- | --- |
|  | Coef. | [95% Conf.Interval] | | Coef. | [95% Conf.Interval] | |
| Liu G | 1.236696 | 1.1881757 | 1.2852162 | 1.4840559 | 1.2838429 | 1.684269 |
| An | 1.236325 | 1.1873472 | 1.2853035 | 1.4824557 | 1.2804048 | 1.6845068 |
| Harth | 1.240569 | 1.191943 | 1.2891957 | 1.4879124 | 1.2875282 | 1.6882967 |
| Abbasi | 1.236148 | 1.1874927 | 1.2848024 | 1.4823096 | 1.2814798 | 1.6831393 |
| Ji | 1.235495 | 1.1870985 | 1.2838922 | 1.4820703 | 1.2831423 | 1.6809983 |
| Gugatschka | 1.241216 | 1.1925294 | 1.2899019 | 1.4874157 | 1.2867936 | 1.6880379 |
| Smedby | 1.239865 | 1.1911161 | 1.2886145 | 1.4854044 | 1.2843668 | 1.6864419 |
| Shen | 1.23987 | 1.1911181 | 1.2886217 | 1.4853867 | 1.2843386 | 1.6864349 |
| Song | 1.235067 | 1.1866517 | 1.2834826 | 1.4785112 | 1.2790596 | 1.6779628 |
| Baris | 1.236174 | 1.1877531 | 1.2845938 | 1.4861141 | 1.2866679 | 1.6855605 |
| Worrillow | 1.236469 | 1.1876134 | 1.285324 | 1.482488 | 1.280892 | 1.6840839 |
| EI-Din | 1.236036 | 1.1875665 | 1.2845057 | 1.4832342 | 1.2832761 | 1.6831923 |
| Capella? G | 1.239022 | 1.1902926 | 1.2877518 | 1.484835 | 1.2838236 | 1.6858466 |
| Zhou RM | 1.235409 | 1.1870123 | 1.2838055 | 1.4809808 | 1.2820491 | 1.6799124 |
| Lou Y | 1.232649 | 1.1842257 | 1.281073 | 1.4635922 | 1.2645692 | 1.6626152 |
| Agalliu | 1.253915 | 1.2046882 | 1.3031421 | 1.4888918 | 1.2871236 | 1.69066 |
| Agalliu | 1.234384 | 1.1859717 | 1.2827969 | 1.4728885 | 1.2735734 | 1.6722035 |
| Moreno V | 1.234575 | 1.1858834 | 1.2832671 | 1.480662 | 1.2796808 | 1.6816431 |
| Hansen RD | 1.240489 | 1.191728 | 1.2892494 | 1.4858149 | 1.284775 | 1.6868548 |
| Wang LL | 1.23153 | 1.183107 | 1.2799524 | 1.4559076 | 1.2575234 | 1.6542917 |
| Mahimkar MB | 1.233956 | 1.1855541 | 1.2823571 | 1.4651943 | 1.2664647 | 1.6639239 |
| Wang Y | 1.233689 | 1.1851791 | 1.2821985 | 1.4771113 | 1.2769586 | 1.677264 |
| Majumder M | 1.236707 | 1.188049 | 1.2853647 | 1.4829401 | 1.2821124 | 1.6837679 |
| Crew | 1.237317 | 1.1881264 | 1.2865076 | 1.4830377 | 1.2802342 | 1.6858412 |
| Jorgensen | 1.238517 | 1.1899766 | 1.2870573 | 1.4872246 | 1.2870725 | 1.6873767 |
| Kuschel | 1.256289 | 1.2050864 | 1.3074921 | 1.4870504 | 1.2782778 | 1.6958231 |
| Lee | 1.235572 | 1.1871598 | 1.2839847 | 1.4823259 | 1.2829416 | 1.6817101 |
| Bernard-Gallon | 1.238082 | 1.1889948 | 1.2871699 | 1.4833351 | 1.2809162 | 1.6857541 |
| Debniak | 1.243853 | 1.1948198 | 1.2928853 | 1.4860801 | 1.2841514 | 1.6880087 |
| Jakubowska | 1.23954 | 1.1909058 | 1.2881734 | 1.486499 | 1.285959 | 1.6870389 |
| Mechanic | 1.245622 | 1.1964523 | 1.2947911 | 1.4862608 | 1.2839078 | 1.6886138 |
| Mechanic | 1.235955 | 1.1874636 | 1.2844454 | 1.4826801 | 1.2825875 | 1.6827728 |
| Shen | 1.23858 | 1.1900749 | 1.2870845 | 1.4888957 | 1.2889899 | 1.6888014 |
| Smith | 1.235097 | 1.1864682 | 1.2837257 | 1.4810177 | 1.280284 | 1.6817514 |
| Smith | 1.234343 | 1.1859425 | 1.2827424 | 1.4688271 | 1.2699693 | 1.6676849 |
| Zhang | 1.240106 | 1.1915491 | 1.2886621 | 1.4894553 | 1.2894292 | 1.6894814 |
| Hussien | 1.227521 | 1.1790509 | 1.2759902 | 1.4539088 | 1.2560264 | 1.6517911 |
| Jelonek | 1.232051 | 1.1835445 | 1.280558 | 1.4729625 | 1.273011 | 1.6729141 |
| Wang | 1.211738 | 1.1621137 | 1.2613626 | 1.4762548 | 1.2731997 | 1.6793101 |
| Zhou | 1.235421 | 1.1870253 | 1.2838172 | 1.4811387 | 1.2822404 | 1.680037 |
| Sakoda | 1.244588 | 1.1954947 | 1.2936815 | 1.4861401 | 1.2840183 | 1.688262 |
| Qian | 1.235017 | 1.1866008 | 1.283432 | 1.4781449 | 1.2786927 | 1.677597 |
| Yin | 1.23479 | 1.1863936 | 1.2831855 | 1.4728068 | 1.273978 | 1.6716356 |
| Raaschou-Nielsen | 1.238946 | 1.1901284 | 1.2877636 | 1.4842741 | 1.2829006 | 1.6856477 |
| Chang | 1.232832 | 1.1843778 | 1.281287 | 1.4708686 | 1.2712381 | 1.6704991 |
| Chang | 1.235836 | 1.1874183 | 1.2842536 | 1.4840461 | 1.2845954 | 1.6834968 |
| Yin | 1.235434 | 1.1870376 | 1.2838295 | 1.4812994 | 1.2824025 | 1.6801963 |
| Lo?pez-Cima | 1.233339 | 1.1846477 | 1.2820307 | 1.4793802 | 1.2784295 | 1.6803309 |
| De Ruyck | 1.235859 | 1.1873915 | 1.2843268 | 1.4826748 | 1.282721 | 1.6826286 |
| Zienolddiny | 1.236589 | 1.1879313 | 1.2852464 | 1.4828073 | 1.2819772 | 1.6836375 |
| Matullo | 1.237345 | 1.1887724 | 1.2859181 | 1.484452 | 1.2840214 | 1.6848825 |
| Hu | 1.233815 | 1.1854132 | 1.2822176 | 1.4640715 | 1.2653818 | 1.6627612 |
| Shen | 1.235217 | 1.1868263 | 1.2836072 | 1.4776945 | 1.2791613 | 1.6762277 |
| Huang | 1.242652 | 1.1937549 | 1.2915483 | 1.4862216 | 1.2847694 | 1.6876738 |
| Broberg | 1.22938 | 1.180928 | 1.2778324 | 1.4560208 | 1.2577072 | 1.6543345 |
| Matullo | 1.236344 | 1.1877186 | 1.2849702 | 1.4826121 | 1.2819055 | 1.6833187 |
| Matullo | 1.238001 | 1.1894486 | 1.286553 | 1.4859403 | 1.285665 | 1.6862158 |
| Schabath | 1.234931 | 1.1862166 | 1.2836448 | 1.4810946 | 1.2800238 | 1.6821654 |
| Andrew | 1.234014 | 1.1853744 | 1.2826536 | 1.4797184 | 1.2789569 | 1.68048 |
| Garcia-Closas | 1.235468 | 1.1863065 | 1.2846295 | 1.4822662 | 1.2795506 | 1.6849817 |
| Wu | 1.234324 | 1.1855129 | 1.2831349 | 1.4808946 | 1.2794598 | 1.6823293 |
| Fontana | 1.235609 | 1.1871816 | 1.2840368 | 1.4822301 | 1.2826144 | 1.6818458 |
| Chang | 1.229041 | 1.180369 | 1.2777122 | 1.4743532 | 1.2738276 | 1.6748787 |
| Gangwar | 1.223016 | 1.1744868 | 1.2715454 | 1.4540986 | 1.2568147 | 1.6513824 |
| Mittal | 1.224392 | 1.175861 | 1.2729224 | 1.4574764 | 1.2595149 | 1.6554378 |
| Ye | 1.235371 | 1.1867979 | 1.2839437 | 1.4812627 | 1.2807628 | 1.6817626 |
| Tse | 1.232446 | 1.1837962 | 1.2810965 | 1.4779253 | 1.2771876 | 1.678663 |
| Pan | 1.235762 | 1.1873584 | 1.2841663 | 1.4845048 | 1.285359 | 1.6836505 |
| Pan | 1.234352 | 1.1856977 | 1.2830062 | 1.4802266 | 1.2793981 | 1.6810553 |
| Huang | 1.235217 | 1.1868263 | 1.2836072 | 1.4776945 | 1.2791613 | 1.6762277 |
| Li | 1.235344 | 1.1869384 | 1.2837486 | 1.4803857 | 1.2811517 | 1.6796197 |
| Han | 1.240325 | 1.1917374 | 1.288913 | 1.4886508 | 1.2884564 | 1.6888452 |
| Han | 1.239548 | 1.1908718 | 1.2882245 | 1.4858693 | 1.285129 | 1.6866095 |
| Han | 1.24083 | 1.1921154 | 1.2895436 | 1.4866253 | 1.2858264 | 1.6874241 |
| Lovatt | 1.243402 | 1.1946535 | 1.292151 | 1.4883312 | 1.2876508 | 1.6890115 |
| Li | 1.237326 | 1.1885222 | 1.2861291 | 1.4831346 | 1.2817529 | 1.6845163 |
| Millikan | 1.268138 | 1.2184634 | 1.3178127 | 1.490121 | 1.2880309 | 1.6922112 |
| Debniak | 1.236698 | 1.1877768 | 1.2856188 | 1.482639 | 1.2808027 | 1.6844752 |
| Bau | 1.231328 | 1.1827539 | 1.2799025 | 1.4746183 | 1.2743421 | 1.6748946 |
| Mandal | 1.227124 | 1.1785839 | 1.2756643 | 1.4646049 | 1.2654753 | 1.6637344 |
| Lavende | 1.231845 | 1.1834185 | 1.2802715 | 1.4595995 | 1.260866 | 1.6583329 |
| Dhillon | 1.236028 | 1.1875784 | 1.2844768 | 1.483706 | 1.2839016 | 1.6835104 |
| Yuan T | 1.235594 | 1.1871109 | 1.2840769 | 1.4817163 | 1.2816573 | 1.6817752 |
| Chen Z | 1.222774 | 1.1743134 | 1.2712353 | 1.4322556 | 1.23857 | 1.6259412 |
| Zhang CZ | 1.229279 | 1.180819 | 1.2777389 | 1.4578291 | 1.259334 | 1.6563243 |
| Ruzzo A | 1.230485 | 1.182019 | 1.2789516 | 1.4637737 | 1.2646393 | 1.6629083 |
| Deng Sl | 1.235811 | 1.1873405 | 1.2842822 | 1.4824748 | 1.2824978 | 1.6824518 |
| Wu | 1.236013 | 1.1874688 | 1.2845562 | 1.4824224 | 1.2820634 | 1.6827812 |
| Sambuddha | 1.231865 | 1.1834393 | 1.2802898 | 1.4592124 | 1.2605168 | 1.6579081 |
| BENJAM?N | 1.236266 | 1.1878345 | 1.2846974 | 1.4857111 | 1.2861148 | 1.6853073 |
| BENJAM?N | 1.237047 | 1.1885891 | 1.2855058 | 1.4872446 | 1.2874769 | 1.6870122 |
| BENJAM?N | 1.237535 | 1.1890759 | 1.2859945 | 1.4889663 | 1.2892725 | 1.6886601 |
| Min Ni | 1.234445 | 1.1860271 | 1.2828622 | 1.4742132 | 1.2747846 | 1.6736419 |
| Combined | 1.235217 | 1.1868264 | 1.2836072 | 1.4776945 | 1.2791614 | 1.6762277 |
